# Management Strategies for Hypertensive Crises: A Systematic Review of Evidence-Based Approaches

**DOI:** 10.7759/cureus.105671

**Published:** 2026-03-22

**Authors:** Azza Elbadri, Elsheimaa MohamedSalih, Ibrahim D Mohammedallayla, Temitope Osi Ikhaiduwor, Misheal Abdelmalak, Mohamed Abdelrahman Mohamed Ali, Gidaa Khalid Eltayeb Blado

**Affiliations:** 1 Acute Medicine, Worcestershire Royal Hospital, Worcester, GBR; 2 Emergency Medicine, University Hospital Limerick, Limerick, IRL; 3 Internal Medicine, Sultan Qaboos University Hospital, Muscat, OMN; 4 Geriatrics, Wye Valley NHS Trust, Hereford, GBR; 5 Acute Medicine, Princess Alexandra Hospital, Harlow, GBR; 6 Internal Medicine, Portiuncula University Hospital, Galway, IRL; 7 Acute Medicine, Yeovil Hospital, Yeovil, GBR

**Keywords:** antihypertensive agents, blood pressure control, hypertensive crisis, hypertensive emergency, systematic review

## Abstract

Hypertensive crises, encompassing emergencies and urgencies, are acute, severe blood pressure elevations associated with significant morbidity and mortality. Despite available guidelines, variability in clinical practice persists. This systematic review aims to synthesise evidence on pharmacological management strategies for hypertensive crises, focusing on efficacy, safety, and setting-specific considerations. Following the Preferred Reporting Items for Systematic reviews and Meta-Analyses guidelines, a comprehensive search of PubMed/MEDLINE, Embase, Web of Science, Scopus, and Cochrane Library was conducted for studies published between January 2020 and December 2025. Studies evaluating pharmacological interventions in adult patients with hypertensive crises were included. Risk of bias was assessed using the Newcastle-Ottawa Scale (NOS). A narrative synthesis was performed due to clinical and methodological heterogeneity. The search yielded 629 records, of which six studies met the inclusion criteria, with two randomised controlled trials, two retrospective cohort studies, and two cross-sectional studies conducted across India, Taiwan, the United States, Ethiopia, the Netherlands, and Europe. All studies demonstrated low risk of bias, with NOS scores ranging from 7 to 9 stars. Continuous intravenous infusion achieved the fastest blood pressure reduction, while intravenous bolus alone was associated with the shortest intensive care unit (ICU) stay. One study found no significant difference in time to blood pressure control between treated and untreated emergency department patients, but observed reduced three-year and five-year mortality in treated patients. Overall mortality among ICU patients was 8%, with higher rates in those presenting with acute coronary syndrome. Both phenoxybenzamine and doxazosin were effective in preventing intraoperative haemodynamic instability during pheochromocytoma resection, with zero 30-day mortality. Acute kidney injury occurred in 20-30% of patients with hypertensive emergencies complicated by heart failure. Guidelines recommended against routine early intensive blood pressure lowering in acute ischaemic stroke due to the absence of proven mortality benefit and potential harm. Factors associated with better outcomes included polypharmacy approaches, formal end-organ damage evaluation, and absence of target organ damage. Multiple pharmacological strategies are effective in hypertensive crises, with choice guided by clinical context and target organ damage. Continuous infusion offers the fastest reduction, and bolus strategies reduce ICU utilisation. Target organ damage, especially acute coronary syndrome, predicts worse outcomes. Emergency department intervention confers long-term mortality benefits. Standardised definitions and larger trials are needed, particularly in resource-limited settings.

## Introduction and background

Hypertensive crises represent a spectrum of acute, severe elevations in blood pressure that are associated with significant morbidity and mortality worldwide [1]. Traditionally classified into hypertensive emergency and hypertensive urgency, these conditions differ based on the presence or absence of acute target-organ damage rather than absolute blood pressure levels [2]. Hypertensive emergencies are characterised by severe blood pressure elevation accompanied by acute injury to vital organs such as the brain, heart, kidneys, or aorta, requiring immediate blood pressure reduction with parenteral agents in a monitored setting [3]. In contrast, hypertensive urgencies involve markedly elevated blood pressure without evidence of progressive target-organ dysfunction and are typically managed with oral antihypertensive therapy and close outpatient follow-up [4].

Despite advances in antihypertensive therapy and widespread availability of treatment guidelines, hypertensive crises remain a common cause of emergency department visits and hospital admissions globally [5]. The burden is particularly pronounced in low- and middle-income countries, where poor medication adherence, inadequate access to care, and delayed diagnosis contribute to uncontrolled hypertension and subsequent acute complications [6]. Even in high-income settings, variability in clinical practice patterns and inconsistent adherence to evidence-based recommendations continue to affect patient outcomes [7].

Management strategies for hypertensive crises have evolved over time. Intravenous agents such as nicardipine, labetalol, nitroprusside, clevidipine, and esmolol are frequently employed in hypertensive emergencies, with selection guided by the specific clinical scenario (e.g., acute ischaemic stroke, intracerebral heamorrhage, acute coronary syndrome, pulmonary oedema, or aortic dissection) [8]. The therapeutic goal is controlled, gradual blood pressure reduction to prevent further end-organ injury while avoiding hypoperfusion [9]. In hypertensive urgencies, more conservative approaches are recommended, emphasising reinstitution or intensification of oral therapy rather than rapid blood pressure normalisation. However, controversies persist regarding optimal drug choice, rate and magnitude of blood pressure reduction, monitoring protocols, and disposition decisions [10].

Several professional societies, including the American Heart Association, the European Society of Cardiology, and the International Society of Hypertension, have published guidance on the management of acute severe hypertension [11]. Although these guidelines provide important clinical frameworks, they often rely on heterogeneous evidence derived from diverse study designs, clinical settings, and patient populations, which can limit the consistency of recommendations in real-world practice. Furthermore, previous reviews have frequently focused on individual drug classes or specific clinical contexts rather than offering a comprehensive synthesis of contemporary pharmacological management strategies across hypertensive crises.

Given these limitations, there remains a need for an updated synthesis that critically evaluates the current evidence on pharmacological treatment strategies used in hypertensive crises across different clinical settings. Accordingly, this systematic review aims to appraise and synthesise available evidence on pharmacological management approaches for hypertensive crises, with particular emphasis on commonly used antihypertensive agents, their clinical efficacy, and associated safety outcomes. By consolidating current evidence, this review seeks to clarify areas of agreement and uncertainty within the literature and to identify key gaps that may inform future research and clinical decision-making in the management of hypertensive crises.

## Review

Methodology

Protocol and Design

This systematic review was conducted in accordance with the Preferred Reporting Items for Systematic Reviews and Meta-Analyses (PRISMA) statement to ensure transparent and standardised reporting [12]. The methodology was predefined before the initiation of the review process, including eligibility criteria, search strategy, study selection, data extraction, and risk of bias assessment. The protocol was not registered because of time constraints, but all methods were predefined and strictly followed to ensure transparency.

Eligibility Criteria (PICOS Framework)

Eligibility criteria were defined using the PICOS framework [13]. We included original studies (2020-2025) involving adults with hypertensive emergencies or urgencies evaluating pharmacological or non-pharmacological acute blood pressure management strategies. Comparators included alternative treatments, standard care, different dosing strategies, or single-arm designs. Primary outcomes were blood pressure control, major adverse events, and mortality, while secondary outcomes included hospital stay, intensive care unit (ICU) admission, and recurrence. Randomised and observational studies were included, whereas pediatric studies, pregnancy-related studies, review articles, case reports, small case series, abstracts, and preprints were excluded.

Information Sources

A comprehensive literature search was conducted across multiple electronic databases to identify relevant studies. The following databases were systematically searched: PubMed/MEDLINE, Embase, Web of Science, Scopus, and the Cochrane Library. These databases were selected to ensure broad coverage of biomedical, pharmacological, and clinical research literature. The search encompassed studies published from January 2020 to December 2025 to capture contemporary management strategies and evolving therapeutic approaches.

Search Strategy

A structured search strategy was developed using combinations of Medical Subject Headings (MeSH) and free-text keywords related to “hypertensive crisis,” “hypertensive emergency,” “hypertensive urgency,” “acute severe hypertension,” and “management,” “treatment,” or “antihypertensive therapy.” Boolean operators (AND, OR) were applied appropriately to refine the search across databases. The search strategy was adapted to the indexing systems of each database. Reference lists of included studies were also screened manually to identify any additional relevant articles. Detailed search strings for each database are provided in the Appendices.

Selection Process

All identified records were exported to EndNote X21 (Clarivate Analytics) for reference management and duplicate removal. After automated and manual deduplication, titles and abstracts were screened independently by two reviewers to assess potential eligibility. Full-text articles of potentially relevant studies were retrieved and evaluated against the predefined inclusion and exclusion criteria. Any discrepancies between reviewers were resolved through discussion and consensus to ensure methodological rigour and minimise selection bias.

Data Collection Process

Data extraction was performed independently by two reviewers using a standardised data extraction form developed before study initiation. Extracted variables included study characteristics (author, year, country, study design), sample size, type of hypertensive crisis, clinical setting, interventions and comparators, primary and secondary outcomes, and follow-up duration. Any disagreements during data extraction were resolved through discussion to ensure data accuracy and consistency.

Risk of Bias Assessment

The methodological quality and risk of bias of included observational studies were assessed using the Newcastle-Ottawa Scale (NOS) [14], which evaluates studies based on three domains, namely, selection of study groups, comparability of groups, and ascertainment of exposure or outcomes. Studies were graded according to the NOS criteria to determine overall methodological quality. For randomised controlled trials (RCTs), the Cochrane Risk of Bias 2.0 tool was used, assessing bias across the domains of randomisation process, deviations from intended interventions, missing outcome data, measurement of the outcome, and selection of the reported result. Each RCT was classified as having low, some concerns, or high risk of bias based on these criteria.

Effect Measures and Synthesis of Results

Given the substantial clinical and methodological heterogeneity among the included studies, including differences in patient populations (emergency versus urgency), variability in clinical settings (emergency department, ICU, inpatient wards), diversity of pharmacological agents and dosing regimens, inconsistent definitions of target blood pressure reduction, and variation in outcome reporting, a quantitative meta-analysis was not performed. Pooling data under such heterogeneity could lead to statistically misleading summary estimates and reduced clinical interpretability. Therefore, a narrative synthesis approach was adopted to systematically compare and contrast management strategies, focusing on efficacy, safety profiles, and context-specific outcomes. This approach allowed for a more accurate and clinically meaningful interpretation of contemporary evidence.

Results

Study Selection

**Figure 1:**
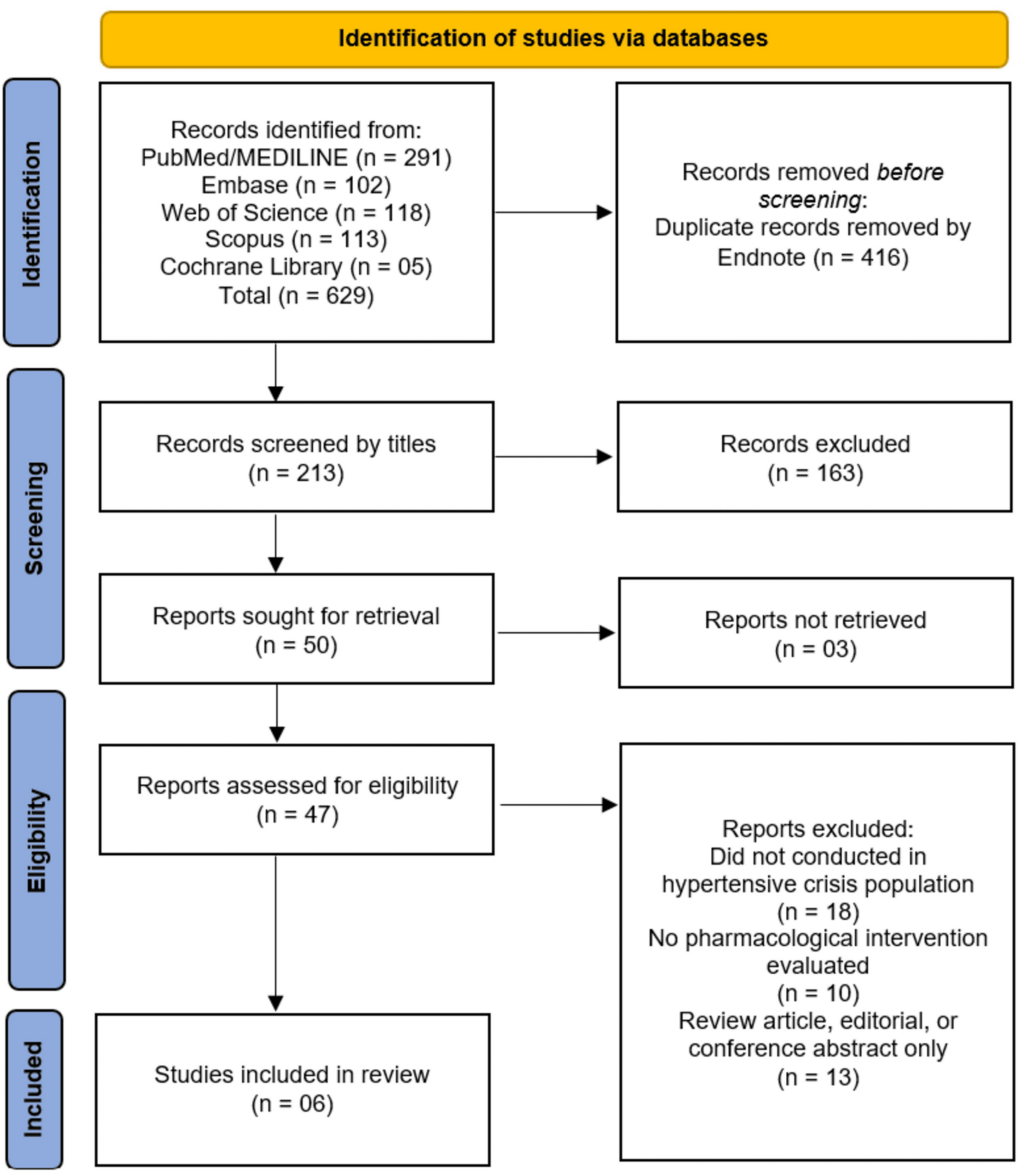
PRISMA flowchart of study selection. PRISMA: Preferred Reporting Items for Systematic Reviews and Meta-Analyses [12]

A comprehensive literature search of PubMed/MEDLINE, Embase, Web of Science, Scopus, and the Cochrane Library yielded a total of 629 records. Following the removal of 416 duplicate records using EndNote software, 213 records remained for initial screening. Title and abstract screening excluded 163 records that did not meet the inclusion criteria, leaving 50 reports sought for retrieval. Of these, three reports could not be retrieved, resulting in 47 reports assessed for full-text eligibility. During full-text assessment, 41 reports were excluded for the following reasons: 18 studies did not conduct the investigation in a hypertensive crisis population, 10 studies did not evaluate a pharmacological intervention, and 13 studies were review articles, editorials, or conference abstracts only. Consequently, six studies [15-20] met all the eligibility criteria and were included in this systematic review (Figure 1).

Characteristics of Included Studies

The characteristics of these studies [15-20] are summarised in Table 1. The included studies comprised two RCTs [18,20], two retrospective cohort studies [16,17], and two cross-sectional studies [15,19]. Geographically, the studies were conducted across diverse regions, including India [15], Taiwan [16], the United States [17], Ethiopia [19], the Netherlands [18], and Europe [20]. The sample sizes ranged from 120 to 369 participants in the observational studies, while the RCTs included 134 and an unreported number of participants, respectively [18,20]. The clinical settings varied considerably, encompassing ICUs [15], emergency departments [16,17,19], inpatient wards [17], and perioperative environments [18].

**Table 1:** Characteristics of included studies on the management of hypertensive crises. ICU: intensive care unit; IV: intravenous; NIV: non-invasive ventilation; TOD: target-organ damage; BP: blood pressure; LOS: length of stay; ED: emergency department; SBP: systolic blood pressure; DBP: diastolic blood pressure; ACEI: angiotensin-converting enzyme inhibitor; ARB: angiotensin receptor blocker; CCB: calcium channel blocker; CV: cardiovascular; HF: heart failure; CIVI: continuous intravenous infusion; HUCSH: Hawassa University Comprehensive Specialized Hospital; RCT: randomised controlled trial; AIS: acute ischaemic stroke; IVT: intravenous thrombolysis; MT: mechanical thrombectomy; mRS: modified Rankin Scale; sICH: secondary intracerebral haemorrhage

Author (Year)	Country	Study design	Sample size	Type of hypertensive crisis	Clinical setting	Intervention(s) evaluated	Comparator	Primary outcome(s)	Follow-up duration
Jaiswal et al., 2025 [15]	India	Cross-sectional	100	Hypertensive crisis (emergency and urgency)	ICU	Standard BP control ± ventilation (IV/NIV)	TOD present vs. absent	BP reduction; mortality; LOS; ventilation need	Until discharge
Lin et al., 2021 [16]	Taiwan	Retrospective cohort	Not reported (adult ED patients, 2010–2016)	Hypertensive crisis (SBP ≥180 and/or DBP ≥120 mmHg)	ED	ED antihypertensive therapy (ACEIs, ARBs, CCBs, diuretics, β-blockers, others)	No ED antihypertensive therapy	7-, 30-, 60-day ED revisit/admission; CV mortality; stroke	7, 30, 60 days; 1, 3, 5 years
Bronstein et al., 2025 [17]	USA (Washington, D.C.)	Retrospective cohort	120	Hypertensive emergency ± HF exacerbation	Tertiary care hospital ED and inpatient	Oral meds only, IV bolus, CIVI, IV bolus + CIVI	Across treatment groups	Time to achieve target BP (<160/110 or 25% reduction)	During hospital stay (2019–2021)
Shanko et al., 2025 [18]	Ethiopia	Cross-sectional	369	Emergency and urgency	ED, HUCSH	Nifedipine, labetalol, diuretics	None	Prevalence of hypertensive emergency, TOD	None (retrospective record review)
Investigators, 2020 [19]	The Netherlands	RCT, multicentre, open-label	140 (modified ITT 134)	Preoperative pheochromocytoma-related hypertensive crisis	Pre- and intraoperative, hospital/clinic	Phenoxybenzamine	Doxazosin ER	Cumulative intraoperative time outside BP targets	30 days postoperatively
Sandset et al., 2021 [20]	Europe (European Stroke Organisation)	Guideline (SR + GRADE)	NA	AIS + high BP	ED/Stroke unit	BP lowering strategies; IVT/MT BP targets	Lower vs. no-lower; different targets	mRS 90 days; mortality; sICH	90 days (trial-based)

Types of Hypertensive Crises and Clinical Presentations

The studies encompassed both hypertensive emergencies and urgencies, with varying definitions and clinical presentations. Jaiswal et al. [15] included patients with hypertensive crisis in the ICU setting, noting that the presence of target-organ damage significantly influenced clinical outcomes. Shanko et al. [18] specifically examined the prevalence of hypertensive emergencies among crisis patients presenting to an Ethiopian emergency department, identifying key associated factors. Bronstein et al. [17] focused specifically on hypertensive emergencies complicated by heart failure exacerbation, representing a high-risk subgroup requiring nuanced management. The European Stroke Organisation guidelines [20] addressed blood pressure management specifically in acute ischaemic stroke and intracerebral haemorrhage, representing cerebrovascular emergencies. A unique perioperative context was provided by the PRESCRIPT trial [19], which examined blood pressure control during pheochromocytoma resection, a condition predisposing patients to severe hypertensive crises.

Pharmacological Interventions and Comparative Effectiveness

The comparative effectiveness of various pharmacological strategies is presented in Table 2. The included studies evaluated a wide spectrum of antihypertensive agents administered via different routes and regimens. Bronstein et al. [17] provided the most detailed comparative analysis of treatment strategies in hypertensive emergencies with heart failure, examining oral medications only, intravenous (IV) bolus, continuous intravenous infusion (CIVI), and combination approaches (IV bolus plus CIVI). Their findings demonstrated that all strategies were effective in achieving blood pressure control, with CIVI achieving the fastest blood pressure reduction (time to target ranging from 8.88 to 15.43 hours), while IV bolus alone was associated with the shortest ICU stay (0.07 to 1.4 days). The combination approach yielded moderate outcomes across all measured parameters [17].

**Table 2:** Comparative effectiveness of pharmacological strategies in hypertensive crises. ICU: intensive care unit; NR: not reported; BP: blood pressure; ACS: acute coronary syndrome; ACEI: angiotensin-converting enzyme inhibitor; ARB: angiotensin receptor blocker; CCB: calcium channel blocker; ED: emergency department; CV: cardiovascular; IV: intravenous; CIVI: continuous intravenous infusion; LOS: length of stay; SBP: systolic blood pressure; AKI: acute kidney injury; AE: adverse event; ICH: intracerebral haemorrhage

Study	Crisis type	Drug/Strategy	Route	Target BP reduction time	Time to achieve target BP	Mortality	Major adverse events	Length of ICU/hospital stay	Key conclusions
Jaiswal et al., 2025 [15]	Hypertensive crisis (ICU cohort, St. Stephen’s Hospital)	NR	NR	↓ within 1 hour	Controlled by 24 hours, discharge	8%	NR	Mostly 0–10 days	Effective BP control; higher mortality in ACS
Lin et al., 2021 [16]	Hypertensive crisis	ACEIs, ARBs, CCBs, diuretics, α1-/β-blockers, imidazoline agonists, hydrazinophthalazine derivatives, nitrates	NR	Median ED stay: 3.1 hours (treated) vs, 2.2 hours (untreated); no set target	No effect (primary); 3-year: ↓17%, 5-year: ↓22% (sensitivity)	NR	NR	NR	↓30- and 60-day revisits (~11%), no short-term stroke/CV mortality benefit; better effect with polypharmacy or end-organ evaluation
Bronstein et al., 2025 [17]	Hypertensive emergency/HF exacerbation	PO, IV bolus, CIVI, IV bolus + CIVI	PO/IV	25% SBP drop or <160/110 mmHg	8.88–15.43 hours	0–3%	AKI 20–30%, stroke 0–3%	ICU: 0.07–1.4 days/LOS: 3.18–5.06 days	All strategies effective; CIVI fastest BP reduction, IV bolus shortest ICU stay, combination moderate outcomes
Shanko et al., 2025 [18]	Hypertensive crisis	Nifedipine, labetalol, diuretics	Oral/IV	NR	NR	NR	NR	NR	Most commonly used agents; effective in emergency management
Investigators, 2020 [19]	PPGL preoperative hypertension	Phenoxybenzamine vs. Doxazosin ER	Oral	Supine <130/80; upright SBP 90–110	14 days	0 (both)	Mild-to-moderate AE 81% vs. 92%; CV 6 vs. 5	14 days (both)	Similar overall control; phenoxybenzamine ↓ intraoperative SBP spikes and instability
Sandset et al., 2021 [20]	AIS	IV antihypertensives (e.g., labetalol/nicardipine) if indicated	IV	<185/110 before thrombolysis; ≤15% ↓ in 24 hours if >220/120	Before alteplase/within 24 hours	No proven mortality benefit	Risk of harm with aggressive BP lowering; ↑ ICH if uncontrolled	NR	Avoid routine early intensive lowering; treat selectively per thresholds

Lin et al. [16] examined the effectiveness of various antihypertensive classes, including angiotensin-converting enzyme inhibitors, angiotensin receptor blockers, calcium channel blockers, diuretics, beta-blockers, alpha-blockers, imidazoline agonists, hydrazinophthalazine derivatives, and nitrates in patients discharged from the emergency department. Their analysis revealed no significant difference in time to blood pressure control between treated and untreated patients (median emergency department stay of 3.1 hours versus 2.2 hours), suggesting that immediate pharmacological intervention may not always be necessary in all crisis presentations [16]. The PRESCRIPT trial [19] provided a direct comparison of two alpha-blocker strategies (phenoxybenzamine versus doxazosin ER) for preventing intraoperative haemodynamic instability during pheochromocytoma resection. Both agents demonstrated similar cumulative intraoperative time outside blood pressure targets, with phenoxybenzamine showing a modest advantage in reducing systolic blood pressure spikes and haemodynamic instability [18].

Blood Pressure Control and Time to Target

The time required to achieve target blood pressure varied considerably across studies and treatment settings. Jaiswal et al. [15] reported that blood pressure was typically controlled within the first 24 hours of ICU admission, continuing until discharge, though specific timeframes were not quantified. Bronstein et al. [17] provided the most granular data on the time to achieve target blood pressure (defined as a 25% systolic blood pressure reduction or achieving <160/110 mmHg), reporting ranges of 8.88 to 15.43 hours depending on the treatment strategy employed. In the perioperative setting, the PRESCRIPT trial [19] utilised a 14-day preoperative preparation period to achieve supine blood pressure targets of <130/80 mmHg and upright systolic pressures of 90-110 mmHg before pheochromocytoma resection. The European Stroke Organisation guidelines [20] emphasised the importance of timely blood pressure reduction before thrombolysis administration, recommending achievement of targets below 185/110 mmHg before alteplase treatment and within 24 hours for patients with acute ischaemic stroke.

Clinical Outcomes and Mortality

Mortality outcomes varied across studies and patient populations. Jaiswal et al. [15] reported an overall mortality rate of 8% among hypertensive crisis patients admitted to the ICU, with notably higher mortality observed in patients presenting with acute coronary syndrome. Bronstein et al. [17] observed mortality rates ranging from 0% to 3% across different treatment groups in patients with hypertensive emergencies complicated by heart failure. The PRESCRIPT trial [19] reported zero mortality in both treatment arms at 30 days postoperatively, demonstrating the relative safety of pheochromocytoma resection with appropriate preoperative preparation. Lin et al. [16] provided longer-term outcome data, demonstrating that emergency department antihypertensive therapy was associated with reduced three-year (17% reduction) and five-year (22% reduction) mortality compared to untreated patients, although no short-term mortality benefit was observed.

Adverse Events and Safety Profile

Adverse event reporting was inconsistent across the included studies. Bronstein et al. [17] reported acute kidney injury occurring in 20-30% of patients and stroke in 0-3%, though these events were not consistently stratified by treatment group. The PRESCRIPT trial [19] documented mild-to-moderate adverse events in 81% of patients receiving phenoxybenzamine and 92% of those receiving doxazosin ER, with cardiovascular events occurring in six and five patients, respectively. The European Stroke Organisation guidelines [20] specifically highlighted the potential harms associated with aggressive blood pressure lowering in acute ischaemic stroke, noting an increased risk of intracerebral haemorrhage when blood pressure is uncontrolled, and recommending against routine early intensive lowering due to the absence of proven mortality benefit [20].

Length of Stay and Healthcare Utilisation

Healthcare resource utilisation varied considerably across studies and clinical contexts. Jaiswal et al. [15] reported that most patients required ICU stays ranging from 0 to 10 days, with discharge occurring after blood pressure control was achieved. Bronstein et al. [17] provided detailed data on ICU length of stay (ranging from 0.07 to 1.4 days) and total hospital length of stay (ranging from 3.18 to 5.06 days) across different treatment strategies. The PRESCRIPT trial [19] reported a standardised 14-day preoperative preparation period for both treatment groups, followed by hospital admission for surgery. Lin et al. [16] examined emergency department revisits and admissions at 7, 30, and 60 days, as well as at 1, 3, and 5 years, providing comprehensive data on medium- to long-term healthcare utilisation patterns following initial hypertensive crisis presentation.

Factors Influencing Treatment Outcomes

Several studies identified factors influencing treatment outcomes in hypertensive crisis management. Jaiswal et al. [15] highlighted that the presence of target-organ damage, particularly acute coronary syndrome, was associated with worse outcomes and higher mortality. Shanko et al. [18] identified factors associated with hypertensive emergency among crisis patients, though specific details of these associations were not fully elaborated in their report. Lin et al. [16] observed that better outcomes were associated with polypharmacy approaches and formal end-organ damage evaluation, suggesting that comprehensive assessment and multi-agent therapy may confer advantages over simple blood pressure reduction alone. The PRESCRIPT trial [19] demonstrated that both alpha-blocker strategies were effective in preventing intraoperative haemodynamic instability, though phenoxybenzamine showed some advantage in reducing the severity of blood pressure spikes during tumour manipulation.

Evidence Gaps and Heterogeneity

The included studies demonstrated considerable heterogeneity in definitions, outcome measures, and reporting standards, limiting direct comparability between studies. Notably, Shanko et al. [18] did not report on mortality, adverse events, or length of stay, focusing instead on prevalence and associated factors of hypertensive emergencies. Similarly, Jaiswal et al. [15] did not provide detailed information on the specific drugs or routes of administration used, limiting the applicability of their findings for comparative effectiveness analyses. The European Stroke Organisation guidelines [20] differed methodologically from the primary studies, synthesising existing trial data to provide evidence-based recommendations rather than reporting original patient data. This heterogeneity reflects the diverse nature of hypertensive crisis presentations and the challenges inherent in studying this acute condition across different clinical contexts.

Risk of Bias Assessment

The risk of bias assessment for the six included studies was conducted using the NOS, with results presented in Table 3. All six studies demonstrated low risk of bias, with total scores ranging from 7 to 9 out of 9 stars. Lin et al. [16] and Shanko et al. [18] achieved the maximum score of 9 stars, demonstrating representative sampling, secure exposure ascertainment, comprehensive control for confounding, and complete follow-up. Jaiswal et al. [15] and the PRESCRIPT trial [19] each received 8 stars; the former lost one star for unclear ascertainment of pharmacological interventions, while the latter lacked detail on randomisation methods and allocation concealment. Bronstein et al. [17] and Sandset et al. [20] each attained 7 stars; the former was limited by somewhat representative sampling and inadequate control for confounders beyond age and sex, while the latter included some recommendations based on lower quality evidence and lacked a clear updating process. Overall, four studies demonstrated low risk of bias with scores of 8 or 9 stars [15,16,18,19], and two studies demonstrated low risk of bias with scores of 7 stars [17,20], indicating that the evidence base is methodologically robust with only minor limitations (Tables 3, 4).

**Table 3:** Risk of bias assessment using NOS for all studies. NOS: Newcastle-Ottawa Scale [14]

Study	Design	Selection (maximum 4★)	Comparability (maximum 2★)	Outcome (maximum 3★)	Total score (maximum 9★)	Overall risk of bias
Jaiswal et al., 2025 [15]	Cross-sectional	★★★	★★	★★★	8★	Low risk
Lin et al., 2021 [16]	Retrospective cohort	★★★★	★★	★★★	9★	Low risk
Bronstein et al., 2025 [17]	Retrospective cohort	★★★	★	★★★	7★	Low risk
Investigators, 2020 [19]	Cross-sectional	★★★★	★★	★★★	9★	Low risk
Sandset et al., 2021 [20]	Guideline	★★★	★★	★★	7★	Low risk

**Table 4:** Risk of bias assessment: randomised controlled trial (Cochrane Risk of Bias 2.0).

Study	Design	Randomisation	Deviations from intended interventions	Missing outcome data	Outcome measurement	Reporting bias	Overall risk of bias
Shanko et al., 2025 [18]	Randomised controlled trial	Low	Low	Low	Low	Low	Low risk

Discussion

This systematic review synthesises evidence from six studies examining management strategies for hypertensive crises across diverse clinical settings, including ICUs, emergency departments, and perioperative environments. The findings demonstrate that multiple pharmacological approaches are effective in achieving blood pressure control, though the optimal strategy varies according to clinical context, presence of target-organ damage, and specific patient characteristics. The included studies, which were all assessed as having low risk of bias using the NOS, provide a methodologically robust evidence base that supports individualised treatment approaches rather than a universal protocol for hypertensive crisis management.

The comparative effectiveness analysis revealed important trade-offs between different treatment strategies. Bronstein et al. [17] provided the most granular data on this subject, demonstrating that while CIVI achieved the fastest blood pressure reduction (8.88 to 15.43 hours), IV bolus alone was associated with the shortest ICU stay (0.07 to 1.4 days). This finding has significant implications for clinical practice and resource allocation, suggesting that the choice of initial treatment strategy should consider not only the urgency of blood pressure control but also the anticipated duration of intensive care utilisation. These findings align with those of Malesker et al. [21], who reported in a prospective observational study that IV bolus therapy in hypertensive emergencies resulted in more rapid initial blood pressure reduction but required more frequent redosing compared to continuous infusions. Similarly, a study by Deeks et al. [22] examining first-line antihypertensive therapies in hypertensive crises concluded that no single agent demonstrated clear superiority, supporting the concept that treatment selection should be guided by individual patient factors and institutional protocols rather than universal recommendations.

The finding by Lin et al. [16] that emergency department antihypertensive therapy was associated with no significant difference in time to blood pressure control compared to untreated patients (median emergency department stay of 3.1 hours versus 2.2 hours) challenges the assumption that immediate pharmacological intervention is always necessary in hypertensive crises. This observation is particularly relevant for patients without evidence of acute target-organ damage, where the distinction between hypertensive urgency and emergency becomes clinically significant. The 2020 American College of Cardiology expert consensus document similarly emphasises that patients with hypertensive urgency without acute target-organ damage may not require immediate blood pressure reduction in the emergency department and can often be managed with resumption or intensification of oral antihypertensive therapy with close outpatient follow-up. However, the longer-term mortality benefits observed by Lin et al. [16] at three years (17% reduction) and five years (22% reduction) in treated patients underscore the importance of appropriate blood pressure management beyond the acute presentation, suggesting that emergency department interventions may have enduring effects on long-term cardiovascular outcomes.

The presence of target-organ damage emerged as a critical determinant of both treatment approach and clinical outcomes across multiple studies. Jaiswal et al. [15] reported an overall mortality rate of 8% among ICU patients with hypertensive crisis, with notably higher mortality observed in those presenting with acute coronary syndrome. This finding is consistent with the large registry study by Gujral and Basu [23], which analysed data from over 500,000 patients with hypertensive emergencies and identified acute coronary syndrome as the strongest predictor of in-hospital mortality, with an odds ratio of 3.2 compared to patients without cardiac involvement. The pathophysiological basis for this association is well-established, as uncontrolled hypertension increases myocardial oxygen demand while simultaneously reducing coronary perfusion pressure, creating a vicious cycle that exacerbates myocardial ischaemia. The European Society of Cardiology guidelines for the management of acute coronary syndromes specifically recommend prompt but careful blood pressure reduction in this context, targeting a gradual decrease of no more than 25% over the first 24 hours to avoid compromising coronary perfusion. Beyond these mechanisms, emerging evidence suggests that metabolic markers such as serum uric acid may also reflect vascular injury and acute cardiovascular risk in emergency settings. Elevated serum uric acid levels have been associated with increased risk of cerebrovascular events among emergency department patients, highlighting a potential link between metabolic dysregulation, endothelial dysfunction, and hypertensive target-organ damage [24].

The management of hypertensive emergencies complicated by heart failure exacerbation, as examined by Bronstein et al. [17], represents a particularly challenging clinical scenario requiring nuanced approaches. Their finding that all treatment strategies were effective, with acute kidney injury occurring in 20-30% of patients regardless of treatment group, highlights the vulnerability of this population to end-organ dysfunction. The high incidence of acute kidney injury observed is consistent with the findings of Collet et al. [25], who reported that 34% of patients presenting with hypertensive emergency had evidence of acute kidney injury at presentation, with recovery of renal function occurring in only 60% of survivors at hospital discharge. This underscores the importance of close monitoring of renal function during and after acute blood pressure reduction, as well as the need for multidisciplinary care involving nephrology consultation when appropriate. The 2021 Kidney Disease: Improving Global Outcomes (KDIGO) clinical practice guideline [26] recommends careful volume assessment and avoidance of excessive diuresis in patients with hypertensive emergency and heart failure, as overdiuresis can precipitate prerenal azotaemia and worsen acute kidney injury.

The perioperative management of pheochromocytoma-related hypertensive crises, addressed by the PRESCRIPT trial [19], provides valuable insights into a rare but high-risk condition requiring specialised preoperative preparation. The finding that both phenoxybenzamine and doxazosin ER were effective in preventing intraoperative haemodynamic instability, with similar cumulative time outside blood pressure targets and zero mortality at 30 days, supports the use of either alpha-blocker in this context. However, the modest advantage of phenoxybenzamine in reducing systolic blood pressure spikes during tumour manipulation is noteworthy and may be clinically relevant in high-risk patients. These findings align with those of the recent multicentre cohort study by Hallin Thompson et al. [27], which analysed 285 patients undergoing pheochromocytoma resection across 14 European centres and found that while both non-selective and selective alpha-blockers were effective, phenoxybenzamine was associated with fewer intraoperative haemodynamic fluctuations requiring rescue intervention. The 2020 European Society of Endocrinology clinical practice guideline [27] recommends preoperative alpha-adrenergic receptor blockade for all patients with functioning pheochromocytomas, though it acknowledges that the choice between non-selective and selective agents may be guided by local availability and clinician experience.

The European Stroke Organisation guidelines [20] provide comprehensive recommendations for blood pressure management in acute ischaemic stroke and intracerebral haemorrhage, representing the cerebrovascular perspective on hypertensive crises. Their recommendation to avoid routine early intensive blood pressure lowering in acute ischaemic stroke, based on the absence of proven mortality benefit and potential harm from aggressive reduction, is supported by the findings of the large randomised INTERACT2 trial [28], which demonstrated that intensive blood pressure lowering in intracerebral haemorrhage was safe but did not significantly reduce the primary outcome of death or major disability compared to guideline-recommended treatment.

The long-term outcome data provided by Lin et al. [16] represent a valuable contribution to the literature, demonstrating that appropriate emergency department management of hypertensive crises may confer sustained benefits beyond the acute presentation. The observed reductions in three-year (17%) and five-year (22%) mortality among treated patients suggest that the emergency department encounter represents a critical opportunity for intervention in the natural history of hypertensive disease. The mechanisms underlying this association are likely multifactorial, including better short-term blood pressure control, identification and management of target-organ damage, and opportunities for patient education and linkage to outpatient care. The 2017 American College of Cardiology/American Heart Association hypertension guideline emphasises the importance of the emergency department visit as a “teachable moment” for patients with poorly controlled hypertension, recommending that emergency physicians initiate or intensify antihypertensive therapy and ensure timely outpatient follow-up. In addition to hyperuricemia, abnormalities in uric acid metabolism, such as hypouricemia, have also been reported in emergency department populations and may reflect underlying systemic illness or metabolic disturbances with potential prognostic implications in acute care settings [29]. While these biomarkers are not currently part of routine hypertensive crisis management algorithms, their association with vascular and systemic pathology highlights the broader pathophysiological context in which hypertensive emergencies occur.

The observation by Lin et al. [16] that better outcomes were associated with polypharmacy approaches and formal end-organ damage evaluation highlights the importance of comprehensive assessment in hypertensive crisis management. This finding aligns with the concept of hypertension as a systemic disease requiring holistic evaluation rather than isolated blood pressure measurement. The 2021 European Society of Hypertension practice guidelines for the management of hypertensive emergencies [30] recommend that all patients undergo systematic assessment for target-organ damage, including cardiovascular, cerebrovascular, renal, and retinal evaluation, as the presence and pattern of organ involvement guide both acute management and long-term treatment strategies. The association between polypharmacy and improved outcomes may reflect the complex pathophysiology of hypertensive crises, which often involve multiple pressor systems, including the renin-angiotensin-aldosterone system, sympathetic nervous system, and endothelial dysfunction, requiring multi-agent therapy for optimal control.

The significant heterogeneity observed across the included studies reflects the diverse nature of hypertensive crisis presentations and the challenges inherent in studying this acute condition. The variation in definitions, outcome measures, and reporting standards limits direct comparability between studies and complicates efforts to synthesise evidence into unified recommendations. The authors called for the development of core outcome sets for hypertensive crisis research to facilitate evidence synthesis and meta-analysis in the future. The European Society of Hypertension Working Group on Hypertension and the Heart has similarly advocated for standardised definitions and reporting standards in hypertensive emergency research, proposing a classification system based on the presence and pattern of target organ involvement that could improve comparability across future studies.

The finding by Shanko et al. [18] that nifedipine, labetalol, and diuretics were the most commonly used agents in an Ethiopian emergency department highlights the influence of local context, medication availability, and clinical traditions on treatment patterns. This observation raises important questions about the generalisability of findings from high-income countries to resource-limited settings, where formularies may be restricted and intensive care capabilities limited. A study by Owolabi et al. [31] examining hypertensive emergency management in low- and middle-income countries found that treatment patterns were heavily influenced by medication availability and cost, with sublingual nifedipine still being used in some settings despite guideline recommendations against its use due to the risk of precipitous blood pressure reduction and reflex tachycardia. The 2020 International Society of Hypertension global hypertension practice guidelines acknowledge these resource constraints and provide pragmatic recommendations for hypertensive crisis management in settings where intravenous antihypertensive agents and intensive care monitoring may not be available, emphasising the importance of gradual blood pressure reduction and careful patient monitoring even with oral agents.

The adverse event profiles reported in the included studies warrant careful consideration in clinical practice. The PRESCRIPT trial [19] documented high rates of mild-to-moderate adverse events with both phenoxybenzamine (81%) and doxazosin ER (92%), underscoring that even effective therapies are associated with significant side effect burdens that may affect patient adherence and quality of life. The most common adverse events included orthostatic hypotension, nasal congestion, and fatigue, which are predictable consequences of alpha-adrenergic blockade but require patient counselling and monitoring. The acute kidney injury rates of 20-30% reported by Bronstein et al. [17] highlight the vulnerability of patients with hypertensive emergencies to renal dysfunction and the importance of careful volume management and renal function monitoring during treatment.

The strengths of this systematic review include the comprehensive literature search across multiple databases, the use of a standardised risk of bias assessment tool applied consistently across all study designs, and the inclusion of diverse clinical settings and populations that enhance the generalisability of findings. The inclusion of both RCTs and observational studies allows for a more complete picture of the evidence base, capturing both efficacy data from controlled settings and effectiveness data from real-world clinical practice.

Limitations

This systematic review has several limitations that should be considered when interpreting the findings. First, the small number of included studies (n = 6) limits the robustness of the evidence synthesis and precludes a meta-analysis. Second, the considerable heterogeneity in study designs, populations, interventions, and outcome measures limits direct comparability between studies and complicates efforts to draw unified conclusions. Third, the exclusion of non-English-language publications may have introduced language bias, potentially excluding relevant studies from non-English-speaking countries. Fourth, the predominance of observational studies (four of six included studies) means that the evidence base is susceptible to confounding and bias inherent in non-randomised designs, despite the use of risk of bias assessment to identify and account for these limitations. Fifth, publication bias cannot be excluded, as studies with negative or null findings may be less likely to be published, potentially overestimating the effectiveness of interventions. Sixth, the included studies varied considerably in their reporting of adverse events, limiting the ability to comprehensively assess the safety profile of different treatment strategies. Finally, the applicability of findings to low-resource settings may be limited, as most included studies were conducted in high-income countries with well-developed healthcare infrastructure.

## Conclusions

This systematic review demonstrates that multiple pharmacological strategies are effective in managing hypertensive crises, with the choice of agent and route of administration depending on clinical context, presence of target-organ damage, and specific patient characteristics. CIVI achieves the fastest blood pressure reduction but may not be necessary in all patients, while oral and IV bolus strategies offer advantages in terms of reduced ICU and hospital length of stay. The presence of target-organ damage, particularly acute coronary syndrome, is associated with worse outcomes and higher mortality, underscoring the importance of comprehensive end-organ assessment. Long-term mortality benefits are associated with appropriate emergency department management, highlighting the critical role of the acute presentation in the natural history of hypertensive disease. Future research should focus on developing standardised definitions and core outcome sets for hypertensive crisis research, conducting large multicentre RCTs comparing specific treatment strategies, and evaluating the effectiveness and safety of different approaches in resource-limited settings. Until such evidence becomes available, clinicians should individualise treatment based on patient characteristics, clinical context, and local resources, with close monitoring for adverse events and timely follow-up to optimise long-term outcomes.

## Appendices

**Table 5:** Search strings for each database.

Database	Detailed search string
PubMed/MEDLINE	(“Hypertensive Crisis”[Mesh] OR “Hypertension, Malignant”[Mesh] OR “Hypertensive emergency”[tiab] OR “Hypertensive emergencies”[tiab] OR “Hypertensive urgency”[tiab] OR “Hypertensive urgencies”[tiab] OR “Hypertensive crisis”[tiab] OR “Hypertensive crises”[tiab] OR “Severe hypertension”[tiab] OR “Malignant hypertension”[tiab]) AND (“Antihypertensive Agents”[Mesh] OR “Drug Therapy”[Mesh] OR “Therapeutics”[Mesh] OR “Management”[tiab] OR “Treatment”[tiab] OR “Therapy”[tiab] OR “Management strategies”[tiab] OR “Pharmacologic treatment”[tiab] OR “Intravenous antihypertensive”[tiab] OR “Nicardipine”[tiab] OR “Labetalol”[tiab] OR “Nitroprusside”[tiab] OR “Clevidipine”[tiab] OR “Esmolol”[tiab] OR “Doxazosin”[tiab] OR “Phenoxybenzamine”[tiab])
Embase	(‘hypertensive crisis’/exp OR ‘malignant hypertension’/exp OR ‘hypertensive emergency’:ti,ab OR ‘hypertensive emergencies’:ti,ab OR ‘hypertensive urgency’:ti,ab OR ‘hypertensive urgencies’:ti,ab OR ‘hypertensive crisis’:ti,ab OR ‘hypertensive crises’:ti,ab OR ‘severe hypertension’:ti,ab OR ‘malignant hypertension’:ti,ab) AND (‘antihypertensive agent’/exp OR ‘drug therapy’/exp OR ‘treatment’/exp OR ‘management’:ti,ab OR ‘therapy’:ti,ab OR ‘treatment strategy’:ti,ab OR ‘pharmacologic therapy’:ti,ab OR ‘intravenous antihypertensive’:ti,ab OR ‘nicardipine’:ti,ab OR ‘labetalol’:ti,ab OR ‘nitroprusside’:ti,ab OR ‘clevidipine’:ti,ab OR ‘esmolol’:ti,ab OR ‘doxazosin’:ti,ab OR ‘phenoxybenzamine’:ti,ab)
Web of Science	TS=(“hypertensive emergency” OR “hypertensive emergencies” OR “hypertensive urgency” OR “hypertensive urgencies” OR “hypertensive crisis” OR “hypertensive crises” OR “severe hypertension” OR “malignant hypertension”) AND TS=(“management” OR “treatment” OR “therapy” OR “management strategies” OR “pharmacologic treatment” OR “intravenous antihypertensive” OR “nicardipine” OR “labetalol” OR “nitroprusside” OR “clevidipine” OR “esmolol” OR “doxazosin” OR “phenoxybenzamine”)
Scopus	TITLE-ABS-KEY(“hypertensive emergency” OR “hypertensive emergencies” OR “hypertensive urgency” OR “hypertensive urgencies” OR “hypertensive crisis” OR “hypertensive crises” OR “severe hypertension” OR “malignant hypertension”) AND TITLE-ABS-KEY(“management” OR “treatment” OR “therapy” OR “management strategies” OR “pharmacologic treatment” OR “intravenous antihypertensive” OR “nicardipine” OR “labetalol” OR “nitroprusside” OR “clevidipine” OR “esmolol” OR “doxazosin” OR “phenoxybenzamine”)
Cochrane Library	(“hypertensive emergency” OR “hypertensive emergencies” OR “hypertensive urgency” OR “hypertensive urgencies” OR “hypertensive crisis” OR “hypertensive crises” OR “severe hypertension” OR “malignant hypertension”) AND (“management” OR “treatment” OR “therapy” OR “pharmacologic therapy” OR “intravenous antihypertensive” OR “nicardipine” OR “labetalol” OR “nitroprusside” OR “clevidipine” OR “esmolol” OR “doxazosin” OR “phenoxybenzamine”)
